# Increased postoperative myeloperoxidase concentration associated with low baseline antioxidant capacity as the risk factor of delirium after cardiac surgery

**DOI:** 10.1080/07853890.2022.2039405

**Published:** 2022-02-17

**Authors:** Jakub Kaźmierski, Piotr Miler, Agnieszka Pawlak, Hanna Jerczyńska, Karina Nowakowska, Grzegorz Walkiewicz, Katarzyna Woźniak, Michał Krejca, Mirosław Wilczyński

**Affiliations:** a Department of Old Age Psychiatry and Psychotic Disorders, Medical University of Lodz, Lodz, Poland; b Central Clinical Hospital, Medical University of Lodz, Lodz, Poland; c CoreLab Central Scientific Laboratory of Medical University of Lodz, Medical University of Lodz, Lodz, Poland; d Department of Cardiac Surgery, Central Clinical Hospital, Medical University of Lodz, Lodz, Poland

**Keywords:** Delirium, cardiac surgery, coronary-artery bypass graft surgery, myeloperoxidase, antioxidant capacity

## Abstract

**Background:**

Though risk factors of postoperative delirium are well described, its pathophysiology is still undiscovered. The primary objective of the current study is to assess whether increased pre- and postoperative myeloperoxidase (MPO) levels are associated with postoperative delirium in the population of cardiac surgery patients. The secondary objective is to evaluate the correlation between MPO levels and serum antioxidant capacity (AC).

**Methods:**

The patients’ cognitive status was assessed one day preoperatively with the use of the Mini-Mental State Examination Test and the Clock Drawing Test. A diagnosis of major depressive disorder and anxiety disorders was established based on DSM-5 criteria. Blood samples for MPO and AC levels were collected both pre- and postoperatively. The Confusion Assessment Method for the Intensive Care Unit was used to screen for a diagnosis of delirium.

**Results:**

Delirium occurred in 34% (61 of 177) of patients. Multivariable logistic regression analysis revealed that increased postoperative MPO concentration was independently associated with postoperative delirium development, and negatively correlated with lower baseline serum AC.

**Conclusions:**

Cardiac surgery patients with less efficient antioxidative mechanisms experience a higher postoperative peak of serum MPO, which in turn may predispose to postoperative delirium development.

**KEY MESSAGES**
- MPO is a lysosomal enzyme with strong pro-oxidative and pro-inflammatory properties.
- Cardiac surgery patients who have increased concentration of postoperative MPO are at significantly higher risk of postoperative delirium development.
- This higher level of postoperative MPO is negatively correlated with baseline antioxidant capacity (AC).
- It can be hypothesized that individuals with decreased baseline AC experience a higher peak of MPO post-surgery due to less efficient antioxidative mechanisms, which in turn contributes to postoperative delirium development.

## Introduction

Coronary-artery bypass graft (CABG) surgery improves cardiac function and patients’ prognosis; however, on the other hand is associated with high risk of developing neuropsychiatric symptoms including postoperative delirium [1]. Post-cardiac surgery delirium occurs in 16–52% of patients; however, reported incidence varies and depends on methodology and diagnostic tools used [2–4]. Delirium has long-term consequences including increased mortality and morbidity, increased risk of falls and dementia development, and decreased functional status [4]. Though delirium risk factors are well described, its pathophysiology is still poorly identified. Myeloperoxidase (MPO) is a lysosomal enzyme found in azurophilic granules of neutrophil granulocytes and monocytes and is characterized by powerful pro-oxidative and pro-inflammatory properties [5]. MPO activity plays a crucial role not only in the physiology of host defence against microorganisms but also in the pathophysiology of atherosclerosis, respiratory tract and central nervous system diseases [6]. Myeloperoxidase has been proposed as a useful risk marker and diagnostic tool in acute coronary syndromes and patients admitted to an emergency room for chest pain [7], as increased serum levels of MPO are predictors of major adverse cardiac events [8]. In addition, correlations between increased serum MPO levels and impaired cognitive functions were observed among patients with a diagnosis of Alzheimer’s disease (AD) [9]. In another study, high baseline serum MPO levels correlated with worse outcomes in tests assessing executive cognitive function in individuals with high physical activity but not in the control group [10]. According to the authors, strenuous exercising may result in elevated MPO levels due to increased oxygen intake and excretion of pro-inflammatory mediators inducing host tissue damage via oxidative stress. The primary objective of the current study is to assess whether increased pre- and postoperative MPO levels are associated with postoperative delirium in the population of cardiac surgery patients. The secondary objective is to evaluate the correlation between MPO levels, and pre- and postoperative serum antioxidant capacity (AC) in the study population.

## Methods

### Study population and eligibility criteria

The procedures and methodology of the current study regarding anaesthesia and surgery, collection of blood samples, statistical analysis and postoperative delirium assessment were previously used in the study of our design, and described elsewhere [11,12]. The study was approved by the Ethics Committee of the Medical University of Lodz, Poland; approval number RNN/95/17/KE. The procedures used in the study were under ethical standards of the Declaration of Helsinki. The adult individuals scheduled for elective cardiac surgery between April 2017 and November 2019 were eligible for participation in the study. The patients were included if they signed informed consent and were presented for isolated CABG surgery or CABG surgery with cardiac valve replacement (CVR). Patients with CABG underwent both on-pump (with cardiopulmonary bypass (CPB)) and off-pump (without CPB) surgery; however, the impact of CPB on the risk of postoperative delirium was controlled in the statistical analysis. The exclusion criteria were as follows: concomitant surgery other than CABG or CABG with CVR; preoperative delirium; active alcohol or other substances addiction (abstinence period shorter than 3 months); illiteracy; pronounced hearing and/or visual impairment. The participants were recruited consecutively.

### Neuropsychiatric assessment

Patients’ cognitive status was evaluated with the use of Mini-Mental State Examination (MMSE) and the Clock Drawing Test (CDT) the day before the scheduled operation [13,14]. The Diagnostic and Statistical Manual of Mental Disorders (DSM-5) criteria were used for major depressive disorder (MDD) and anxiety disorders diagnosis [15].

### Anaesthesia

For induction of anaesthesia fentanyl 5–10 mcg/kg, propofol 1–2.5 mg/kg and rocuronium 0.6–1.0 mg/kg were used. During maintenance phase, fentanyl in continuous intravenous infusion in doses of 2–10 mcg/kg/h, propofol 3–10 mg/kg/h, interrupted doses of rocuronium were administered. Ventilation was provided with a breathing mixture of FiO_2_ 0.5 and air to maintain end-tidal CO_2_ at 35–45 mmHg. From surgical incision to CPB connection sevoflurane 0.5–2 vol% was used.

Following surgical intervention, the patients were transferred to the ICU where mechanical ventilation was continued. Until extubation, morphine in the continuous infusion of 1–2 mg per hour and propofol perfusion at a rate 1–2 mg/kg/h were used for sedation. The criteria for extubation were as follows: arterial blood gases and oxygen saturation >92% and stabilization of hemodynamic parameters.

### Surgery

Patients who underwent CABG or CABG with concomitant valve surgery were operated through median sternotomy and on CPB under normothermia. The anterograde DelNido cardioplegia was used in all patients during the operation. In some cases, patients who underwent CABG were operated without CPB (off-pump CABG), on a beating heart, either through the median sternotomy or through left-sided mini-thoracotomy.

### Measurement of serum MPO concentration and antioxidant capacity

The venous blood samples were collected the day prior to the surgery and on the first postoperative day, between the hours 07:00 and 09:00 a.m. The samples were centrifuged at 7000 rpm for 10 min and were refrigerated at −80 °C until the biomarkers analysis. For proteins measurement, commercially available ELISA kits and an Antioxidant Assay Kit (Cayman Chemical, Ann Arbor, MI) were used. Protocols were performed following the manufacturer’s instructions. The concentration of proteins in the samples was determined by interpolation from the standard curve. The absorbance was read by Multifunctional Microplate Reader VICTORTM X4 (Perkin Elmer, Waltham, MA). All ELISA results were analysed with WorkOut 2.5 Software and the mean concentration of protein per mL was determined by referring to the four-parameter logistic (4-PL) curve. For washing steps, the Stat-Matic Plate Washer II (Sigma-Aldrich, St. Louis, MO) was used. The investigators who performed the laboratory analysis were blinded to the patients’ clinical data.

### Delirium diagnosis

The patients were screened for delirium with the use of algorithm of the Confusion Assessment Method for the Intensive Care Unit (CAM-ICU) and Memorial Delirium Assessment Scale (MDAS) with the cut-off score of 10 [16,17].

The treatment team was trained on delirium symptoms recognition and asked to inform the clinician if any change in patients’ cognition, consciousness or behaviour occurred. Delirium screening of each patient was done once a day within the first five days after surgery and in case of any change in patients’ mental and/or behavioural condition reported by the treatment team.

### Statistical analysis

For categorical variables, the number of observations (*n*) and a fraction (%) are calculated, whereas quantitative variables are expressed as medians and interquartile ranges (IQRs).

The normality was assessed using Shapiro–Wilk’s test. Differences between two independent samples for continuous data were analysed with the use of Mann–Whitney’s *U*-test (since the distributions of variables were different from normal). The effect size for continuous variables was evaluated with the rank-biserial correlation coefficient.

Differences between two independent samples for categorical data were assessed with the chi-squared test or Fisher’s exact test. The effect size for categorical variables was calculated with Cramer’s *V* coefficient. The minimum study sample size was calculated using the power analysis, estimating the expected effects from our previous studies and assuming an alpha level of 0.10 and a power of 80% (minimum sample size for each group is 37 patients). Initially, baseline, intra- and postoperative variables were evaluated for univariate association with postoperative delirium. For quantitative variables (preoperative and postoperative MPO concentration), significantly associated with delirium, receiver operating characteristic (ROC) curves were created and decision thresholds were found. The analysis of sensitivity, specificity, positive predictive value and negative predictive value of the found thresholds were calculated. Factors significant in univariate comparisons (*p*<.10) were included in a forward stepwise logistic regression model to identify the set of the independent risk factors for delirium. The results were considered significant for *p*<.05. All of the calculations were performed using STATISTICA (version 13.3, 2017; StatSoft, Inc., Tulsa, OK).

## Results

During the observational period, 294 patients were eligible for the study. There were 70 subjects not included in the study due to the following reasons: surgery different than CABG/CABG combined with CVR (*n* = 58); refusal to participate (*n* = 12). Of 224 patients who signed informed consent and were enrolled, four patients were lost to follow-up since they died before observational period was completed, and 43 individuals had incomplete study data.

The final analysis included 177 patients with a median age 67 years (IQR: 63–71). Delirium was diagnosed in 61 (34%) of 177 enrolled subjects. The results of the univariate analysis of baseline and perioperative variables are shown in Tables 1–3.

**Table 1.** Baseline characteristics in univariate comparisons.

Variable	Non-delirious^a^ (*n* = 116)	Delirious^a^ (*n* = 61)	Effect size^b^	*p*
Age (years)	66 (61–69)	70 (66–72)	−0.340	<.001
Gender female	15 (13.0%)	24 (39.0%)	0.303	<.001
Depression	9 (7.8%)	24 (39.0%)	0.385	<.001
Anxiety disorders	5 (4.3%)	9 (14.7%)	0.184	.02
Alcohol addiction	8 (6.9%)	5 (8.2%)	0.024	.768
Peripheral vascular disease	13 (11.2%)	17 (27.9%)	0.211	.005
Arterial hypertension	89 (76.7%)	56 (91.8%)	0.186	.013
NYHA	2 (2–2)	2 (2–3)	−0.175	.029
AF^c^	10 (8.6%)	12 (19.7%)	0.159	.034
Diabetes	35 (30.0%)	26 (42.6%)	0.125	.098
Urea concentration (mmol/L)	6.8 (5.5 − 7.6)	6.7 (5.4–8.0)	0.018	.849
Creatinine concentration (mmol/L)	83.7 (75.4–98.3)	88 (68.1–104.8)	−0.028	.758
Anaemia^c^	16 (13.8%)	11 (18.0%)	0.056	.456
Cerebrovascular disease	12 (10.3%)	9 (14.7%)	0.065	.464
COPD	6 (5%)	5 (8.25)	0.060	.516
CCS	2 (2–3)	2 (2–3)	0.115	.503

AF: atrial fibrillation; CCS: Canadian Cardiovascular Society Degree; COPD: chronic obstructive pulmonary disease; NYHA: New York Heart Association grade.

^a^ For continuous variables, the medians and interquartile ranges (IQRs) are given.

^b^ For continuous variables, rank-biserial correlation coefficient was calculated; for categorical variables, Cramer’s *V* coefficient was given.

^c^ Haemoglobin concentration <10 mg/dL.

**Table 2.** Perioperative characteristics in univariate comparisons.

Variable	Non-delirious^a^ (*n* = 116)	Delirious^a^ (*n* = 61)	Effect size^b^	*p*
CABG with cardiac valve replacement	8 (6.9%)	9 (14.75%)	0.127	.092
ECC	81 (69.8%)	52 (85%)	0.170	.024
Hyperthermia^d^	9 (7.8%)	10 (16.4%)	0.133	.078
Aortic cross-clamping^c^ (min)	40 (30–55)	43 (30–70)	−0.114	.270
Duration of surgery (h)	4.0 (3–4.5)	4.0 (4–4.5)	−0.085	.350
Circulatory support^c^	2 (1.7%)	1 (1.6%)	0.003	.97
Corticosteroids use^c^	0 (0%)	1 (1.6%)	0.104	.345
pCO_2_≥45^d^ (mmHg)	24 (20.7%)	18 (29.5%)	0.099	.19
pO_2_≤60^d^ (mmHg)	18 (15.5%)	13 (21.3%)	0.072	.33

CABG: coronary-artery bypass graft; ECC: extracorporeal circulation.

^a^ For continuous variables, the medians and interquartile ranges (IQRs) are given.

^b^ For continuous variables rank-biserial correlation coefficient was calculated; for categorical variables Cramer’s *V* coefficient was given.

^c^ Intraoperative variables.

^d^ Postoperative variables.

**Table 3.** Pre- and postoperative biomarkers in univariate comparisons.

Variable	Non-delirious^a^ (*n* = 116)	Delirious^a^ (*n* = 61)	Effect size^b^	*p*
Preoperative MPO (ng/mL)	254.02 (156.36–374.41)	314.05 (216.41–446.21)	−0.193	.035
Postoperative MPO (ng/mL)	330.35 (202.54–451.18)	431.21 (294.5–534.14)	−0.331	<.001
Preoperative AC (µmol/L)	2.38 (1.85–3.08)	1.32 (1.04–2.31)	0.443	<.001
Postoperative AC (µmol/L)	2.11 (1.42–2.94)	1.37 (0.90–1.89)	0.409	<.001

AC: antioxidant capacity; MPO: myeloperoxidase.

^a^ For continuous variables, the medians and interquartile ranges (IQRs) are given.

^b^ For continuous variables, rank-biserial correlation coefficient was calculated; for categorical variables, Cramer’s *V* coefficient was given.

The median preoperative and postoperative MPO concentration in the whole population was 275.9 ng/mL (IQR: 161.5–413.2) and 361.0 ng/mL (IQR: 233.6–488.3), respectively. The median preoperative and postoperative AC levels in the whole population were 2.13 µmol/L (IQR: 1.29–2.91) and 1.84 µmol/L (IQR: 1.22–2.65), respectively.

Univariate analysis showed the patients with increased pre- and postoperative MPO levels had a higher risk of postoperative delirium development comparing to patients with lower MPO concentrations (Table 1). Of note, when controlling for variables significant in univariate comparisons, only patients with higher postoperative MPO levels remained at increased risk of postoperative delirium development (Table 4). In addition, postoperative MPO was negatively correlated with preoperative antioxidant activity (Spearman’s rank correlation coefficients: −0.171; *p*<.05). The correlations between MPO levels and pre- and postoperative AC were not observed (Spearman’s rank correlation coefficients for preoperative MPO and pre- and postoperative AC: −0.144 and −0.044, respectively, *p*>.05; Spearman’s rank correlation coefficients for postoperative MPO and postoperative AC: −0.076; *p*>.05). Other factors independently associated with delirium were: age, gender female, MDD diagnosis, peripheral vascular disease diagnosis and the presence of CPB (Table 4). According to the ROC analysis, the most optimal cut-off value of postoperative MPO levels that predicts the development of delirium was 376.93 ng/mL with sensitivity of 65.6% and specificity of 64.7%, positive predictive value of 0.49 and negative predictive value of 0.78 (OR = 3.48; 95% CI: 1.81–6.68) (area under the curve = 0.66; standard error = 0.042; 95% CI: 0.58–0.75; *p*<.001).

**Table 4.** Multivariable stepwise logistic regression model showing factors independently associated with postoperative delirium.^a^

Variables	OR (95% CI)	*p*
Depression^b^	9.85 (3.56–27.25)	.000
Gender female	5.78 (2.33–14.39)	.000
Age	1.10 (1.04–1.18)	.003
Peripheral vascular disease^b^	3.68 (1.21–11.21)	.024
Extracorporeal circulation	3.43 (1.17–10.11)	.029
Postoperative myeloperoxidase	1.002 (1.000–1.004)	.034
Constant	–	.000

^a^ The regression model is statistically significant: *χ*^2^=41.350, df = 6, *p*<.001; Hosmer–Lemeshow test: *χ*^2^=9.457, *p*=.305; Nagelkerke *R*^2^=0.449.

^b^ Preoperative variables.

## Discussion

This prospective study was composed of 177 adults undergoing cardiac surgery, of whom 34% experienced postoperative delirium. According to univariate analysis, both higher pre- and postoperative MPO levels were associated with post-surgery delirium, albeit, multivariable logistic regression model indicated that only MPO concentration raised after the intervention was independently associated with postoperative delirium.

In available studies, MPO was shown to elicit strong pro-inflammatory and pro-oxidative properties independently of its catalytic activity [7,18]. The relationship between higher cytokines levels (IL-6), C-reactive protein (CRP) and delirium was previously observed in the population of ICU patients and individuals after non-cardiac surgery [19–21]. Moreover, the association between increased neutrophil-to-lymphocyte ratio and postoperative delirium in elderly patients with total hip arthroplasty for hip fracture was reported [22].

Our previous prospective study conducted among cardiac surgery patients revealed that raised levels of IL-2 and TNF-α measured in the postoperative period are associated with the development of delirium among CABG surgery patients [23]. This higher concentration of pro-inflammatory cytokines was additionally associated with longer duration of CPB and related to advancing age and pre-operative cognitive decline. Furthermore, we reported the independent association between preoperatively elevated monocyte chemoattractant protein-1 (MCP-1) concentration and the risk of postoperative delirium development [12].

MPO is an oxidative stress biomarker secreted by leukocytes and macrophages in response to ischemia–reperfusion injuries [24]. High MPO serum levels predict risk for early cardiac events in patients with the acute coronary syndrome, as well as major adverse cardiovascular events (death, nonfatal myocardial infarction and stroke) [8]. According to Origer et al. study (2013), MPO secretion increases significantly during on-pump cardiac surgery, and its values peak just after aortic de-clamping [24]. Since activation of phagocytic leukocytes and neutrophils is crucial for immune response, it can be hypothesized, that MPO activity mediates the mechanisms involved in stress/immune response in the course of cardiac surgery. Leukocytes activation results in the accelerated formulation of an NADPH oxidase (NOX)-2 enzyme complex at the plasma membrane and a subsequent “oxidative burst” and excessive superoxide radicals production. These radicals undergo dismutation (catalysed by superoxide dismutase) to form molecular oxygen and hydrogen peroxide which show bactericidal and cytotoxic effects [7]. Although the generation of oxidants by MPO is beneficial with regard to the immune response to invading pathogens, inappropriate and excessive stimulation of oxidant formation by this enzyme can result in host tissue damage.

In study by Gellhaar et al., the number of MPO-immunoreactive cells was significantly increased in brain areas affected by neurodegeneration, such as the frontal cortex in AD and the caudate, putamen and midbrain in PD [5]. These cells did not resemble or co-localize with markers for astrocytes or neurons but were confined to microglia and located close to blood vessels. In experimental studies, MPO deficiency prevents the development of delayed cognitive impairment (DCI) after subarachnoid haemorrhage. Albeit, re-introduction of biologically active MPO to the mice meninges at the time of the haemorrhage elicited cognitive deficit seen in DCI [25]. Judging from previous studies and current analysis, we may hypothesize that MPO levels which increase significantly after cardiac surgery may cause blood–brain barrier and neurological damage presenting with cognitive deficits including delirium. Importantly, in the current study, patients with lower baseline serum AC had significantly higher postoperative MPO levels. Antioxidant capacity reflects the human antioxidant system activity and consists of such enzymes as superoxide dismutase and catalase, and macromolecules such as albumin and ceruloplasmin to counteract free radicals and prevent tissue damage. MPO has strong pro-oxidative properties. It mediates the formation of hypochlorous acid and hydrogen peroxide, the powerful oxidants which interact with other small molecules including NH_3_ to form monochloramines or with other ROS to yield peroxynitrite (ONOO−) and hydroxyl radical (·OH) [26]. Therefore, it can be hypothesized that individuals with decreased baseline AC experience higher peak of MPO post-surgery due to less efficient antioxidative mechanisms, which in turn contributes to postoperative delirium development.

## Data Availability

The datasets used and/or analysed during the current study are available from the corresponding author on reasonable request.
